# The magnetic susceptibility effect of gadolinium-based contrast agents on PRFS-based MR thermometry during thermal interventions

**DOI:** 10.1186/2050-5736-1-8

**Published:** 2013-06-04

**Authors:** Nicole M Hijnen, Aaldert Elevelt, Jeroen Pikkemaat, Clemens Bos, Lambertus W Bartels, Holger Grüll

**Affiliations:** 1 Biomedical NMR, Department of Biomedical Engineering, Eindhoven University of Technology, High Tech Campus 11.p 261, Eindhoven, 5656 AE, the Netherlands; 2 Department of Minimally Invasive Healthcare, Philips Research Eindhoven, Eindhoven, 5656 AE, the Netherlands; 3 Image Sciences Institute, Imaging Division, University Medical Center Utrecht, Utrecht, 3584 CX, the Netherlands

**Keywords:** Gadolinium, MR thermometry, Susceptibility, MR-HIFU, Image-guided drug delivery

## Abstract

**Background:**

Proton resonance frequency shift (PRFS) magnetic resonance (MR) thermometry exploits the local magnetic field changes induced by the temperature dependence of the electron screening constant of water protons. Any other local magnetic field changes will therefore translate into incorrect temperature readings and need to be considered accordingly. Here, we investigated the susceptibility changes induced by the inflow and presence of a paramagnetic MR contrast agent and their implications on PRFS thermometry.

**Methods:**

Phantom measurements were performed to demonstrate the effect of sudden gadopentetate dimeglumine (Gd-DTPA) inflow on the phase shift measured using a PRFS thermometry sequence on a clinical 3 T magnetic resonance-guided high-intensity focused ultrasound (MR-HIFU) system. By proton nuclear magnetic resonance spectroscopy, the temperature dependence of the Gd-DTPA susceptibility was measured, as well as the effect of liposomal encapsulation and release on the bulk magnetic susceptibility of Gd-DTPA. *In vivo* studies were carried out to measure the temperature error induced in a rat hind leg muscle upon intravenous Gd-DTPA injection.

**Results:**

The phantom study showed a significant phase shift inside the phantom of 0.6 ± 0.2 radians (mean ± standard deviation) upon Gd-DTPA injection (1.0 mM, clinically relevant amount). A Gd-DTPA-induced magnetic susceptibility shift of Δχ_Gd-DTPA_ = 0.109 ppm/mM was measured in a cylinder parallel to the main magnetic field at 37°C. The temperature dependence of the susceptibility shift showed dΔχ_Gd-DTPA_/d*T* = -0.00038 ± 0.00008 ppm/mM/°C. No additional susceptibility effect was measured upon Gd release from paramagnetic liposomes. *In vivo*, intravenous Gd-DTPA injection resulted in a perceived temperature change of 2.0°C ± 0.1°C at the center of the hind leg muscle.

**Conclusions:**

The use of a paramagnetic MR contrast agent prior to MR-HIFU treatment may influence the accuracy of the PRFS MR thermometry. Depending on the treatment workflow, Gd-induced temperature errors ranging between -4°C and +3°C can be expected. Longer waiting time between contrast agent injection and treatment, as well as shortening the ablation duration by increasing the sonication power, will minimize the Gd influence. Compensation for the phase changes induced by the changing Gd presence is difficult as the magnetic field changes are arising nonlocally in the surroundings of the susceptibility change.

## Background

Magnetic resonance-guided high-intensity focused ultrasound (MR-HIFU) is gaining interest as a noninvasive method for thermal therapies ranging from local tumor hyperthermia to thermal ablation. In these procedures, MRI is used for planning of the HIFU treatment as well as to dynamically map HIFU-induced temperature changes [1,2]. Accurate temperature feedback to the ultrasound control unit is a prerequisite to obtain mild tumor hyperthermia over a prolonged period of time, as well as for safety and to provide thermal dose feedback on the tumor coverage during ablation treatment [3].

Several MR temperature monitoring techniques have been proposed [4], of which proton resonance frequency shift (PRFS) thermometry is by far the most commonly used in water-containing tissues [5]. The frequency shift arises from the influence of temperature on the hydrogen bonds between molecules, resulting in a change in the electronic screening of the hydrogen nuclei and therefore the local magnetic field the nuclei experiences [4,6]. Over the temperature range of interest for HIFU therapy, the resulting water proton chemical shift is linearly related to the temperature change with an average value of -0.01 ppm/°C [4,7,8].

The temperature-induced frequency shift is measured by the subtraction of gradient-echo phase images acquired before and during heating. As only a *change* in frequency is measured using the subtraction method, PRFS thermometry measures temperature differences rather than absolute temperatures. Consequently, PRFS thermometry is also sensitive to magnetic field changes of other origin than temperature changes alone [9,10]. Examples of magnetic field changes are drift of the scanner's main magnetic field and field changes caused by changes in the volume magnetic susceptibility distribution inside the subject. The influence of field drift on PRFS thermometry has been widely recognized and can be corrected for by making use of, e.g., a reference region in which no heating is applied [11]. Magnetic susceptibility changes can have various origins. They can, for example, arise from the heating of fat tissue as described by Sprinkhuizen et al. [10,12] or from motion [13]. The demagnetizing effects caused by objects made of a material with a susceptibility different from that of the tissue depend on the shape, i.e., cylinder or sphere, and orientation, i.e., parallel or perpendicular to the main magnetic field, of the object and have been extensively described in the literature [14]. The presence of paramagnetic substances, like MRI contrast agents, also has a strong influence on the magnetic susceptibility. Up to now, the effect of a paramagnetic MRI contrast agent in the tissue on PRFS temperature mapping has not yet been investigated. This effect can be twofold. It can originate, first, from a changing distribution of the paramagnetic contrast agent during PRFS thermometry and, second, from a potential difference in the temperature dependency of the susceptibility of the contrast agent and the tissue.

In MR-HIFU applications, MR contrast agents based on the paramagnetic gadolinium ion (Gd-CA) are commonly used, for example, for tumor delineation during treatment planning and for measuring treatment response [15,16]. Another example is the use of Gd agents encapsulated in temperature-sensitive drug delivery carriers (paramagnetic liposomes) to monitor liposomal content release [17,18]. However, paramagnetic contrast agents such as Gd-CA have a volume magnetic susceptibility that differs considerably from that of the tissue. In the literature, susceptibility-induced frequency shifts, Δχ, of 0.32 ppm/mM for gadopentetate dimeglumine (Gd-DTPA) have been reported. The final effect depends on the geometrical distribution of the contrast agent with respect to the main magnetic field [19-21]. In the clinical practice of contrast-enhanced MRI, local tissue gadolinium concentrations in the low millimolar range are not unusual [22]. The phase differences between images acquired before and during heating with PRFS thermometry will therefore depend not only on the temperature changes but also on changes in the distribution of the Gd agent. As a consequence, PRFS temperature mapping in the presence of gadolinium-containing contrast agents could, without taking proper precautions, lead to local temperature errors of several tens of degrees.

We performed a set of *in vitro* and *in vivo* experiments to measure the effects of Gd-DTPA (MAGNEVIST®, Bayer Schering, Berlin, Germany) on the phase shift measured by PRFS thermometry in different scenarios. First, the effects of a changing concentration of Gd-DTPA on PRFS thermometry were assessed. Next, the temperature dependence of the paramagnetic susceptibility-induced frequency shift of Gd-DTPA was investigated in phantoms. Finally, we investigated whether the release of Gd-CA from paramagnetic temperature-sensitive liposomes had an effect on the bulk magnetic susceptibility.

## Materials and methods

### Phantom measurements: varying Gd-DTPA concentrations

Phantom measurements were performed to demonstrate the effect of sudden change in Gd-DTPA concentration on the phase shift measured using a PRFS thermometry sequence. A plastic cylindrical container (inner diameter = 0.8 cm, outer diameter = 1.0 cm, height = 4.0 cm) containing demineralized water (1.25 mL) was aligned perpendicular to the main magnetic field of a 3 T clinical MR scanner (Philips Achieva®, Best, the Netherlands). The container was positioned and fixed inside a watertight, HIFU-compatible MR receiver coil placed in a larger water pool [23]. The temperature of the fluid inside the container was kept stable and equal to the temperature of the pool water (*T* = 21.2°C). Baseline PRFS temperature mapping was performed for 10 min in the absence of Gd-DTPA using the same PRFS sequence as used for the *in vivo* studies. The sequence was a spoiled gradient echo sequence with an echo planar imaging (EPI) readout (EPI factor, 7) and sensitivity encoding (SENSE) (SENSE factor, 1.8; SENSE direction, right to left) to allow fast imaging. The repetition time (TR) was set to 38 ms with an echo time (TE) of 20 ms. The rest of the PRFS parameters were set as follows: field of view (FOV), 250 × 250 mm^2^; voxel size, 1.4 × 1.4 × 4.1 mm^3^; fat suppression, spectral pre-saturation with inversion recovery (SPIR); number of averages, 4, resulting in a dynamic scan time of 2.1 s. One coronal and one transverse slice were continuously acquired, both through the container midpoint. The evolution of phase *φ* over time was measured directly without further processing. From the phase data, the corresponding temperature change (Δ*T*) was calculated according to [4]

(1) $$\Delta T\left(x,y\right)=\frac{\varphi \left(x,y\right)-\varphi_{0}\left(x,y\right)}{\gamma \alpha B_{0}\mathrm{TE}},$$

where *φ*_0_ is the baseline phase at the start of the measurement (in radians), *γ* the hydrogen gyromagnetic ratio (in radians/second/tesla), *B*_0_ the main magnetic field strength (in tesla), and *α* the temperature coefficient of the water proton electron screening constant (-0.01 ppm/°C) [4]. The corresponding temperature change (i.e., not corrected for the effect caused by a changing Gd distribution) was calculated after the correction of the acquired signal for the drift of the main magnetic field. Therefore, a phase correction was performed by subtracting the average phase in a reference region from the acquired phase image. The reference region was placed inside the water pool, at least 5 cm away from the container.

After baseline measurements were performed, Gd-DTPA (180 μL of 1:50 diluted Magnevist®, [Gd-DTPA]_final_ = 1.0 mM, 20 μL flush) was added to the container using an infusion pump (injection speed = 1 mL/min). The fluid in the container was carefully mixed during a period of 1 min from the start of injection using a Pasteur pipette. Fixation of the container prevented unintended movement during mixing. PRFS thermometry was continued for another 10 min, after which the data were exported and processed in MATLAB (R2010a, MathWorks, Natick, MA, USA). Before and after PRFS thermometry, a map of the longitudinal relaxation time (*T*_1_) was acquired using a steady-state Look-Locker sequence [24,25] (EPI factor, 5; TR/TE, 9.0:3.4 ms; interval time, 100 ms; flip angle *α*, 10°; FOV, 50 × 69 mm^2^; matrix, 64 × 65; fat suppression, SPIR; slice thickness, 5 mm parallel to the cylinder long axis; number of averages, 2; acquisition time, 2:36 min). The effective *T*_1_ (*T*_1_^*^) was calculated from the signal recovery on a voxel-by-voxel basis using an in-house created IDL-based software tool (IDL version 6.3, RSI, Boulder, CO, USA). Further data processing was performed in MATLAB, in which the longitudinal relaxation rate (*R*_1_) was calculated from the effective *R*_1_^*^ (*R*_1_^*^ = 1/*T*_1_^*^, *R*_1_ = *R*_1_^*^ + ln(cos(*α*))/TR, with *α* = 10° and TR = 100 ms [24]) on a voxel-by-voxel basis.

### Phantom measurements: temperature dependence of susceptibility

As the volume magnetic susceptibility of water-containing tissues is known to be temperature dependent, the effect of changing temperature on the water proton shift of a solution containing a stable amount of Gd-DTPA was measured. The proton shifts were measured at three constant Gd-DTPA concentrations (0, 3.13, and 4.67 mM) at five different temperatures (*T* = 37°C, 41°C, 45°C, 50°C, and 55°C) by proton nuclear magnetic resonance (^1^H-NMR) spectroscopy. The Gd-DTPA concentrations were chosen as they approximate the amount of contrast agent present in human malignant breast tumor tissue (Fan et al. [22]), respectively, 30 and 10 min after the injection of the standard clinical Gd-DTPA dose (0.1 mmol/kg bw). Samples containing the desired concentration of Gd-DTPA were prepared by dilution with demineralized water. Five hundred microliters of sample was transferred into a standard thin-walled 5-mm-diameter cylindrical NMR tube (the outer compartment). Before the ^1^H-NMR measurement, a glass capillary (product number: WGS-5BL, Wilmad, Vineland, NJ, USA) containing aqueous (4-(2-hydroxyethyl)-1-piperazineethanesulfonic acid (HEPES) buffer (20 mM, pH 7.5) and 3-(trimethylsilyl)-1-propanesulfonic acid sodium salt (DSS; 1.1 mM) was inserted coaxially into the NMR tube (the inner compartment). Standard ^1^H-NMR spectra were acquired with 128 k complex data points and a dwell time of 41.6 μs using a Bruker AVANCE300 NMR spectrometer (Madison, WI, USA) equipped with a wide-bore 7-T superconducting magnet (Oxford Instruments, Abingdon, UK). In order to prevent line broadening as a result of radiation damping, the 300-MHz RF transmit-receive circuit was detuned by -4 MHz. The peak chemical shift positions of the sample (*δ*_sample_) and reference (*δ*_ref_) were determined using a peak picking algorithm. The frequency of the DSS methyl signal originating from the inner compartment was calibrated for temperature dependence using *δ* = -0.071 - 9.9 × 10^-5^*T* - 5.9 × 10^-7^*T*^2^ (ppm), *T* being the sample temperature (in °C) [26]. The ^1^H-NMR spectra of the diamagnetic reference sample showed a single water signal at a chemical shift moving from 4.6 (37°C) to 4.4 ppm (55°C) upon heating. Apparently, the presence of the small amounts of DSS and HEPES buffer in the coaxial capillary insert did not cause significant differences in the diamagnetic susceptibility. In the paramagnetic spectra, by contrast, two separate water signals were observed: (1) a weak signal at the same frequency as in the corresponding diamagnetic reference spectrum originating from the water in the diamagnetic inner compartment and (2) an intense signal that shifted approximately 0.3–0.5 ppm towards a higher frequency originating from the water in the Gd-DTPA-containing outer compartment (the actual spectra can be found in Additional file 1). The Gd-DTPA-induced frequency shift (in ppm) was obtained from the frequency shift of the water signal between the two measurements, according to the method presented by Corsi et al. (Method 1, [21]). Since Gd-DTPA is not expected to cause a (pseudo)contact shift of the water signal, the susceptibility-induced frequency shift can alternatively be determined from a single NMR spectrum as the frequency difference between the water signals from the paramagnetic and the diamagnetic compartment (Method 2, [25]). Both methods gave identical susceptibility shifts within the experimental error (±0.005 ppm).

### *In vivo* study

The effect of intravenous Gd-DTPA injection on the phase stability in the rat hind limb muscle was measured to demonstrate the effect of Gd-DTPA injection during an *in vivo* study (Fisher 344 rats, *n* = 4). All animal studies were approved by the animal welfare committee of the Maastricht University (the Netherlands) and were in compliance with the guidelines set by the institutional animal care committee, accredited by the National Department of Health.

Ultrasound gel (Aquasonic 100, Parker Laboratories, Fairfield, NJ, USA) was applied onto the shaven leg to minimize tissue-air interfaces. The animal was placed into a dedicated small animal HIFU-compatible MR coil setup (Philips Healthcare, Vantaa, Finland) [23]. The respiration rate of the animals was monitored continuously using a small balloon sensor (Graseby, Smiths Medical, St. Paul, MN, USA). The animal body temperature was monitored using a rectal temperature probe (Neoptix, Québec City, Canada). The body temperature was kept stable by running water through a circuitry positioned below the animal body.

The system stability was assessed by measuring the phase during a period of 4 min prior to Gd-DTPA injection. Gd-DTPA (Magnevist®) was injected via a tail vein catheter using an infusion pump (0.6 mmol/kg bw, 0.5 M, injection speed, 1 mL/min). This dose is the rat equivalent of the human clinical Gd-DTPA dose (0.1 mmol/kg bw) [27]. No HIFU heating was applied. Phase mapping was continued up to 10 min postinjection. The concentration of Gd-DTPA in the muscle tissue was quantified based on the differences in *R*_1_ before and directly after the PRFS measurements. Therefore, *R*_1_ maps of the muscle were acquired using the same Look-Locker protocol as described for the phantom experiments.

### Susceptibility of liposomal encapsulated Gd

Finally, we investigated whether there is an effect of liposomal encapsulation and release on the bulk magnetic susceptibility effect of Gd-CA by ^1^H-NMR spectroscopy. Therefore, the BMS shift (Δ*χ*) of a solution containing paramagnetic temperature-sensitive liposomes (TSLs) was measured before and after temperature-induced contrast agent release using the same spectrometer and method as described in the temperature dependence experiment above. Traditional TSLs were made in-house using a previously described protocol [28]. The aqueous lumen of the TSL contained 250 mM [Gd(HPDO3A)(H_2_O)] (Prohance®, Bracco Diagnostics, Milan, Italy). The final gadolinium concentration of the TSL suspension was 5.3 mM, as determined by inductively coupled plasma-mass spectrometry (ICP-MS; DRCII, PerkinElmer, Waltham, MA, USA). Five hundred microliters of the liposomal suspension was transferred into a standard thin-walled 5-mm-diameter cylindrical NMR tube (the outer compartment). Before the ^1^H-NMR measurement, a glass capillary containing HEPES buffer (20 mM, pH 7.5) and DSS (1.1 mM) was inserted coaxially into the NMR tube (the inner compartment). The effect of the diamagnetic species present in the suspension (phospholipids, sodium chloride, HEPES buffer) on the magnetic susceptibility was assumed negligible [29]. The ^1^H-NMR chemical shift of the water signal in the outer compartment was measured at *T* = 37°C before and after the temperature-induced release of the encapsulated [Gd(HPDO3A)(H_2_O)]. Contrast agent release was effectuated by the heating of the sample to *T* = 50°C for 90 s inside the magnet using the sample heater of the NMR spectrometer and checked by measuring the *R*_1_ of the sample before and after heating using a standard spectroscopic inversion recovery NMR sequence (at 37°C). Inversion-recovery curves (NMR signal intensity versus inversion recovery time) were calculated from the integrals of the water signal. Actual *R*_1_ values were obtained from the inversion-recovery curves using the Levenberg-Marquardt nonlinear least squares fitting procedure of the Bruker TopSpin NMR software. All inversion-recovery curves could be fitted with a pure single-exponential decay function.

## Results

### Phantom measurements: varying Gd-DTPA concentrations

**Figure 1:**
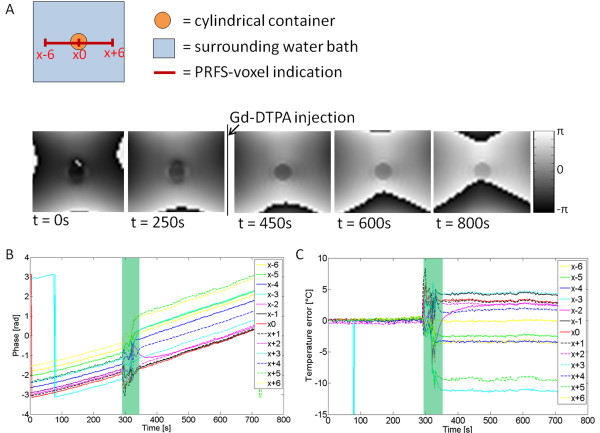
**Varying Gd-DTPA concentrations in phantoms.** (**A**) MRI phase images before and after Gd-DTPA injection. No heating was applied. The *red scale bar* indicates the voxel locations in plots B and C. (**B**) Phase plot in which each *line* indicates the phase over time of a point in the phase image. The *green bar* indicates the time span during which Gd-DTPA was injected and mixed. The phase signal was not corrected for main magnetic field drifts. (**C**) Plot of the calculated temperature error over time. The apparent temperature change was corrected for magnet field drifts and was calculated from the magnetic field changes via the -0.01 ppm/°C relation [4,8].

The effect of a changing amount of Gd-DTPA on the phase measured with a PRFS-based MR thermometry sequence was tested by injecting Gd-DTPA in a water-containing phantom kept at a stable temperature. The phase signal showed a variation over time owing to drift of the main magnetic field (Figure 1B). After correction, a stable baseline temperature profile was observed (Figure 1C). Upon Gd-DTPA injection, the phase signal was heavily disturbed owing to the fast movement of the water molecules through the imaging gradients during the mixing of the contrast agent with the water in the phantom (green bar in Figure 1B,C). After the phantom fluid had stabilized again, the phase shifts after injection were compared with pre-injection and were measured in the middle of the phantom (x0) and in six points to the left and to the right (x-6 to x+6). Phase shift values of 0.6 ± 0.2 radians (mean ± standard deviation (SD)) were observed inside the phantom (x-1 to x+1) comparing before (*t* = 250 s) and after Gd injection (*t* = 400 s). With the used temperature measurement sequence, this translates in a perceived temperature change inside the cylinder of approximately +3.8°C ± 1.3°C. These temperature changes were within the range that was expected from theory based on the Gd-DTPA susceptibility and sample orientation (cylindrical sample oriented perpendicular to the main magnetic field: Δ*χ*_Gd-DTPA_ = 0.32 × (-1/6) = -0.053 ppm/mM, 1 mM Gd-DTPA, -0.053 / -0.01 = +5.3°C maximum PRFS temperature error inside the sample) [30].

### Phantom measurements: temperature dependence of susceptibility

**Figure 2:**
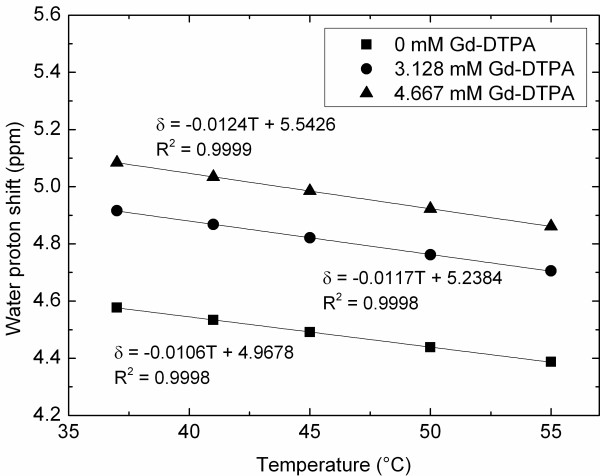
**The Gd-induced susceptibility effect is temperature dependent.** The water proton frequency shift at different temperatures in the presence of different Gd-DTPA concentrations.

Figure 2 displays the water proton chemical shifts at different Gd concentrations and temperatures. The frequency of the water signal (relative to the inner compartment DSS reference signal) in the sample containing demineralized water only (0 mM Gd-DTPA) changed from approximately 4.6 ppm at 37°C to approximately 4.4 ppm at 55°C. A standard fitting procedure with a linear model function yielded excellent fits with a slope of -0.0106 ppm/°C (*R*^2^ = 0.9998). Similar values were previously reported in other studies [4,7,8] and are generally attributed to temperature-dependent variations in the electronic screening of the hydrogen nuclei of water molecules. By subtracting the water proton shift obtained at each temperature with different concentrations of Gd-DTPA, the Δ*χ* induced by the presence of Gd-DTPA and its temperature dependence were calculated. This resulted in Δ*χ*_Gd-DTPA_ = 0.109 ppm/mM at 37°C for a cylinder parallel to the main magnetic field, closely corresponding to values previously found for aqueous Gd chelates [21] and values expected from theory (inside the cylinder parallel to the main magnetic field, 1/3Δ*χ*, 1/3 × 0.32 ppm/mM = 0.107 ppm/mM, which is close to the measured value) [20,29,31]. The temperature dependence measured in the relatively narrow temperature range between 37°C and 55°C showed a linear behavior with dΔ*χ*_Gd-DTPA_/d*T* = -0.00038 ± 0.00008 ppm/mM/°C. More details can be found in Additional file 1.

### *In vivo* study

**Figure 3:**
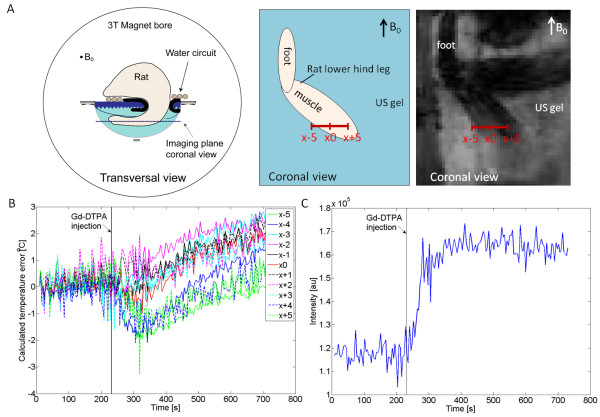
**The effect of Gd injection *in vivo*.** (**A**) Schematic drawings of the animal orientation in transversal and coronal directions, and a magnitude image of the rat hind leg as obtained with the PRFS thermometry sequence (coronal view). The *red scale bar* indicates the voxel locations in plot B. (**B**) The perceived temperature baseline error over time *in vivo* over a horizontal line profile in the rat muscle. (**C**) PRFS voxel signal intensity data obtained at x0 indicating the inflow of Gd-DTPA into the rat hind leg muscle.

Four animals were injected with Gd-DTPA (0.6 mmol/kg bw) to measure the magnetic susceptibility effect of a changing amount of Gd-CA in the tissue on the phase stability of gradient-echo PRFS images. The animal body temperature was tightly regulated and kept constant at 37°C ± 0.2°C, as measured using the rectal temperature probe. *In vivo* PRFS thermometry without heating in the absence of a Gd-based contrast agent showed no average temperature change over a period of 10 min with a standard deviation of 0.3°C. Intravenous Gd-DTPA injection resulted in a change of the local magnetic field, which translated into an apparent temperature change of -2.0°C ± 0.1°C (mean ± SD) in the middle of the hind leg muscle (x0 in Figure 3A). Depending on the PRFS voxel location, the presence of Gd-DTPA in the tissue resulted in either a positive (central area) or a negative (peripheral area) phase shift and apparent temperature change (Figure 3B). As expected based on the literature [14], the susceptibility-induced shift showed a symmetrical pattern around the Gd-DTPA-perfused tissue (i.e., x-5 = x+5, x-4 = x+4, etcetera).

The inflow of Gd-DTPA into the rat hind leg muscle tissue could qualitatively be observed from the magnitude images obtained with the PRFS sequence (Figure 3C). The Gd-DTPA inflow showed coincidence with the observed increase in the perceived temperature error. The amount of Gd-DTPA present in the muscle tissue was quantitatively assessed based on the change in the *R*_1_. For this purpose, *R*_1_ maps were acquired prior to and immediately after PRFS thermometry. Ten minutes after injection, a Δ*R*_1_ ≈ 0.68 s^-1^ was found, indicating that approximately 0.18 mM Gd-DTPA was present in the muscle tissue (assuming Δ*R*_1_ = *r*_1_[CA], with *r*_1_ = 3.7 mM^-1^ s^-1^ (3.5–3.9) the Gd-DTPA relaxivity in plasma at 37°C according to [32]).

### Susceptibility of liposomal encapsulated Gd

^1^H-NMR spectroscopy was performed to investigate whether the liposomal encapsulation of Gd-CA has an influence on the magnetic susceptibility. The proton resonance frequency of the compartment containing paramagnetic liposomes was shifted 0.565 ppm towards a higher frequency compared with the compartment without liposomes. The gadolinium concentration as calculated from this shift Δ*χ* was 5.35 mM, corresponding to the concentration as determined by ICP-MS (5.3 mM). After heating above the liposomal phase transition temperature, inducing Gd release (Δ*R*_1_ ≈ 2 s^-1^), the resonance frequency of the TSL compartment showed a minor additional shift of 0.005 ppm upwards (<1% of the total effect of introducing paramagnetic liposomes). However, similar shifts (between 0.003 and 0.007 ppm) were observed after heating and cooling down of a solution containing [Gd(HPDO3A)(H_2_O)] only (no liposomes) and after heating and cooling down of pure water. Therefore, the frequency shift observed upon Gd release from the liposomes was regarded within the error of detection.

## Discussion

MR-HIFU is increasingly used for noninvasive thermal therapies ranging from mild hyperthermia to thermal ablation. Accurate MR thermometry is crucial to perform such procedures in a controlled and successful manner. PRFS thermometry is the most frequently used MR thermometry method, which uses local nuclear magnetic field changes to calculate temperature differences. Subtraction-based PRFS thermometry is sensitive not only to temperature-induced changes of the electronic screening of water protons (*α* ≈ -0.01 ppm/°C), but also to other magnetic field-influencing effects such as a Gd-based contrast agent flowing in or out of the monitored region and the temperature dependency of the magnetic susceptibility of the tissue with such a contrast agent. The latter effects - if active - cause a systematic error on the experimental PRFS temperature change that has been seriously neglected thus far. Here, we discuss the impact of Gd-DTPA presence on the size and the direction of the PRFS temperature errors.

In the absence of heating, we measured a Gd-induced phase shift in the rat hind leg muscle that would erroneously be interpreted as a 2° temperature difference. These results nicely corresponded to theory (0.18 mM Gd-DTPA in muscle tissue, Δ*χ* = 0.18 × 0.109 ppm/mM = 0.02 ppm maximum shift in the case of cylindrically shaped sample oriented parallel to the main magnetic field, 0.02 / -0.01 = -2.0°C PRFS temperature error). However, in most MR-HIFU cases, the temperature change measurement will not be performed during the injection of the contrast agent or closely thereafter. Obviously, the smaller the change in contrast agent concentration and distribution, the smaller the effect the contrast agent will have on PRFS temperature monitoring. The local changes in contrast agent concentration over time will differ for different tissue types, malignant versus benign tumors, and species (half-life of Gd-DTPA approximately 96 min in humans [33] compared with 19.6 min in rats [34]). Therefore, it is advisable to characterize the behavior and washout of the contrast agent in the area of interest once at the beginning of a new study. Subsequently, the washout information needs to be taken into consideration when deciding on the final MR-HIFU treatment workflow.

To give an indication of the expected temperature error range in human breast cancer treatment, the values measured for human Gd-DTPA clearance rates in malignant breast tumors as described and modeled by Fan et al. [22] were used. Based on their model, the contrast agent concentration in the tumor was calculated at different time points after the injection of a standard clinical Gd-DTPA dose (0.1 mmol/kg bw). The expected Gd-induced water proton shift was calculated based on the contrast agent variations in combination with the measured Gd-DTPA susceptibility (Δ*χ*_Gd-DTPA_ = 0.327 ppm/mM at 37°C) and assuming a spherical tumor [30]. With a perfect spherical geometry, no field shift occurs inside the tumor; however, at the tumor border zone, a Gd-induced shift can be expected of 2/3Δ*χ*_Gd-DTPA_ = 0.218 ppm/mM. Assuming 0.5–3 min per sonication (i.e., the time that the ultrasound transducer is on, assuming a new reference phase map is acquired (*φ*_0_) at the start of each sonication), 30–60 min of waiting time between the injection of the paramagnetic contrast agent and the start of the HIFU treatment, the Gd-induced temperature error at the tumor edges ranges between 0°C and -4°C.

In order to minimize this effect of the contrast agent, it is advised to perform the ablation with a high sonication power (within the safety limits), so that the time per sonication is kept as short as possible. Furthermore, trapping of the contrast agent inside the ablated tissue owing to local vascular shutdown is likely to further reduce the contrast agent change.

The practical implications of the effect caused by the temperature dependence of the magnetic susceptibility of Gd-DTPA (dΔ*χ*_Gd-DTPA_/d*T* = -0.00038 ± 0.00008 ppm/mM/°C, measured in water) are more complicated. Here, the temperature dependence of the magnetic susceptibility of Gd-DTPA was measured in water and not in actual tissue. This is not fully correct considering the temperature-dependent variations that occur in the tissue density and variations between different tissue types. Despite this limitation, we observed that with low amounts (<1 mM) of Gd-DTPA present, the Gd-DTPA magnetic susceptibility closely follows that of pure water and glandular tissue [9,10], leading to no significant changes in the susceptibility distribution upon heating. However, 30 min after the injection of a standard imaging dose of Gd-DTPA, the local amount of Gd-DTPA in human malignant breast tissue approximates 3 mM [22], resulting in a local susceptibility temperature dependence of Δ*χ* ≈ -0.0011 ppm/°C. This value is approximately 10% of that of the electronic screening constant used for the PRFS temperature measurement. Thus, even without Gd-DTPA concentration changes, the susceptibility temperature dependence within the tissue can lead to temperature errors of maximally +3°C in the tumor, during a temperature increase of Δ*T* = 30°C. Errors of this magnitude will influence the accuracy of the thermal dose calculations and thus the expected treatment success. The effect induced by the Gd presence will be retained longer by the slower clearance of the Gd-DTPA owing to the tissue ablation.

Accurate temperature measurements to allow feedback control of the power emitted by the HIFU transducer is also essential to obtain prolonged MR-HIFU-induced hyperthermia, e.g., required for temperature-induced drug delivery (≈30 min at 41°C). Noninvasive MR imaging and quantification of the HIFU hyperthermia-induced drug delivery process has been demonstrated using paramagnetic TSLs co-encapsulating drugs and MRI contrast agents [17,18,35-37]. In this process, the contrast agent is released from the liposomes during heating. Liposomal encapsulation lowers the relaxivity of the Gd-CA owing to reduced water exchange over the liposomal membrane. Upon heating above the liposomal phase transition temperature, the normal relaxivity of the contrast agent is retrieved as water access is restored. The Gd-induced bulk magnetic susceptibility effect, however, occurs at a more macroscopic scale. We measured whether the bulk magnetic susceptibility changes upon the release of the contrast agent from the liposomes. A frequency difference of 0.565 ppm was observed between the signal originating from pure water and the signal coming from the paramagnetic liposomes. Upon heating and release, an additional frequency increase of 0.005 ppm was observed (<1%); however, this shift was also observed after the heating of free Gd-DTPA or pure water, indicating that there was no effect of Gd release from the paramagnetic liposomes itself upon the bulk magnetic susceptibility. Although the Gd release in itself does not cause an additional susceptibility effect, it is crucial in the MR-HIFU hyperthermia-induced drug delivery workflow to perform the paramagnetic liposome injection prior to the acquisition of the reference phase scan (*φ*_0_), which is at the onset of PRFS thermometry. The long blood circulation time (≈2 h) of the paramagnetic liposomes is beneficial as the Gd concentration in the blood will not change as rapid as it does after the injection of un-encapsulated Gd-DTPA.

## Conclusion

Using a paramagnetic contrast agent during MR-HIFU treatments has the advantage to better delineate the ablation target region or to show the release of drug from temperature-sensitive liposomes, but has the disadvantage to influence the accuracy of the PRFS thermometry. Owing to the contrast agent injection, the temperature change calculated based on the phase change becomes a function of the variation in spatial temperature distribution and of the local Gd concentration. Depending on the treatment workflow, Gd-induced temperature errors ranging between -4°C and +3°C can be expected. These errors originate not only from the change in Gd concentration, but also from the different susceptibility temperature dependence of the tissue with and without Gd. Longer waiting time between contrast agent injection and HIFU treatment, as well as shortening the ablation duration by increasing the sonication power, will minimize the Gd influence. Compensation for the phase changes induced by the changing Gd presence is difficult as the magnetic field changes are arising both locally and nonlocally in the surroundings of the susceptibility change.

## Competing interests

The authors AE, JP, and HG are employed by Philips. NMH, CB, LWB, and HG are currently receiving a grant (grant 05T-201) from the Center for Translational Molecular Medicine (http://www.ctmm.nl), project VOLTA.

## Authors’ contributions

NMH carried out the phantom measurements and *in vivo* studies, analyzed and interpreted the data, and drafted the manuscript. AE carried out the phantom measurements, interpreted the data, and participated in drafting the manuscript. JP carried out the spectroscopy measurements and participated in the interpretation of the data. CB and LWB participated in the alignment of the manuscript and critically reviewed it. HG participated in the design and coordination and helped draft the manuscript. All authors read and approved the final manuscript.

## Supplementary Material

Additional file 1 The file contains supplemental Figures S1 to S5 and Table S1. Click here for file
