# Supplementary material for: The magnetic susceptibility effect of gadolinium-based contrast agents on PRFS-based MR thermometry during thermal interventions
Source: J Ther Ultrasound. 2013 Jun 4;1:8. doi: 10.1186/2050-5736-1-8 (PMC4265976; doi:10.1186/2050-5736-1-8)
Supplement: Additional file 1 — The file contains supplemental Figures S1 to S5 and Table S1. [file 2050-5736-1-8-S1.pdf]

SUPPLEMENTAL INFORMATION

The magnetic susceptibility effect of gadolinium-based contrast agents on PRFS-based MR thermometry during thermal interventions

Nicole M Hijnen[1*], Aaldert Elevelt[2*], Jeroen Pikkemaat[2], Clemens Bos[3], Lambertus W. Bartels[3], Holger Gruell[1,2].

[*] *Both authors contributed equally to this paper*
[1] *Biomedical NMR, Department of Biomedical Engineering, Eindhoven University of Technology, the Netherlands*
[2] *Department of Minimally Invasive Healthcare, Philips Research Eindhoven, the Netherlands*
[3] *Image Sciences Institute/Department of Radiology, University Medical Center Utrecht, the Netherlands.*

Phantom measurements: temperature dependence of susceptibility

The ^{1}H-NMR spectra measured for the three Gd-DTPA concentrations (0 mM, 3.13 mM, and 4.67 mM) at each temperatures (T = 37 °C, 41 °C, 45 °C, 50 °C, and 55 °C) are shown in Supplemental figures 1-3.

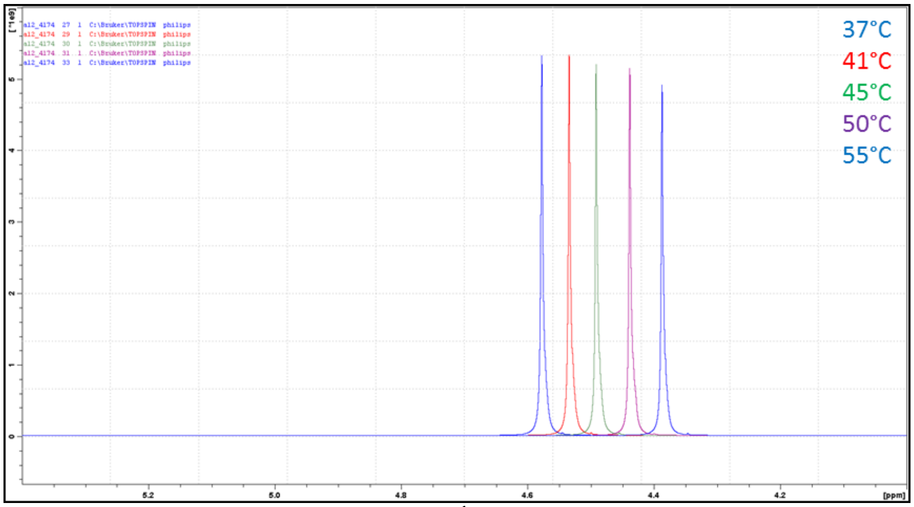

Supplemental figure 1: Overlay of the ^{1}H-NMR spectra of water at five different temperatures.

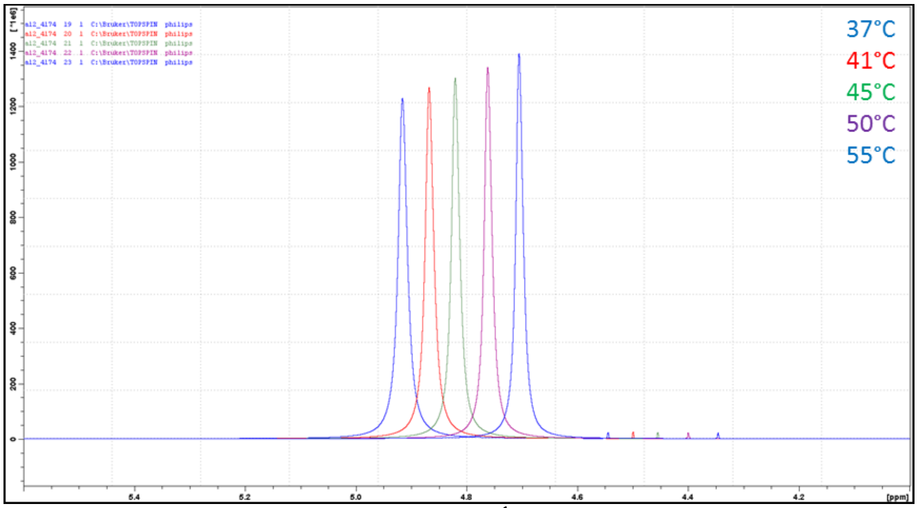

Supplemental figure 2: Overlay of the ^{1}H-NMR spectra of the 3.128 mM Gd-DTPA sample at five different temperatures. The five peaks with a high intensity shifted towards higher frequency originated from the water in the Gd-DTPA containing outer compartment. The low intensity signals originated from the water protons in the diamagnetic inner compartment.

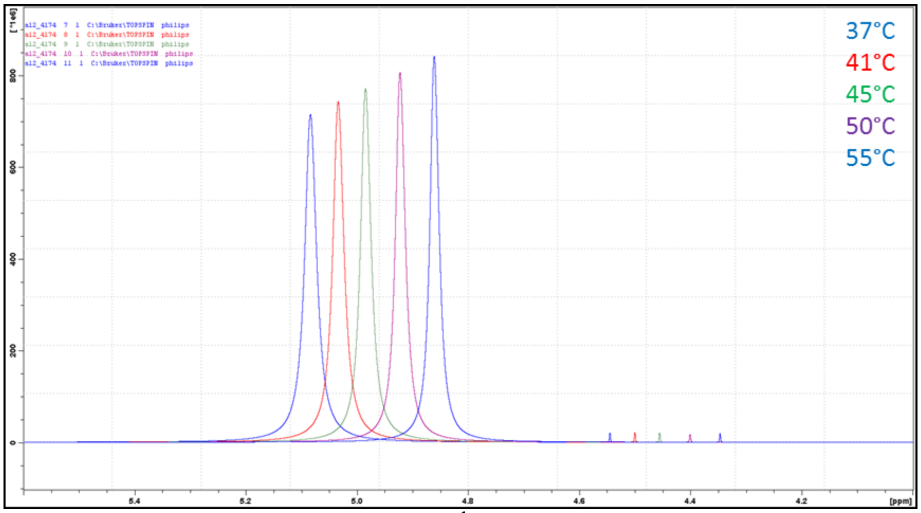

Supplemental figure 3: Overlay of the ^{1}H-NMR spectra of the 4.667 mM Gd-DTPA sample at different temperatures. The five peaks with a high intensity shifted towards higher frequency originated from the water in the Gd-DTPA containing outer compartment. The low intensity signals originated from the water protons in the diamagnetic inner compartment.

Supplemental table 1: Chemical shift positions (δ) of the water signal at different temperatures and Gd-DTPA concentrations. From the change in chemical shift positions, the BMS shift per mM Gd-DTPA was calculated.

Sample temperature (°C)	Sample temp-erature (K)	δ (ppm) pure water	δ (ppm) 3.128 mM Gd-DTPA	BMS shift (ppm) 3.128 mM Gd-DTPA	BMS shift per mM (ppm/mM) 3.128 mM data	δ (ppm) 4.667 mM Gd-DTPA	BMS shift (ppm) 4.667 mM Gd-DTPA	BMS shift per mM (ppm/mM) 4.667 mM data
37.0	310.15	4.5773	4.9162	0.339	0.108	5.0844	0.507	0.109
41.0	314.15	4.5337	4.8678	0.334	0.107	5.0341	0.500	0.107
45.0	318.15	4.4911	4.8208	0.330	0.105	4.9848	0.494	0.106
50.0	323.15	4.4380	4.7622	0.324	0.104	4.9227	0.485	0.104
55.0	328.15	4.3870	4.7054	0.318	0.102	4.8612	0.474	0.102

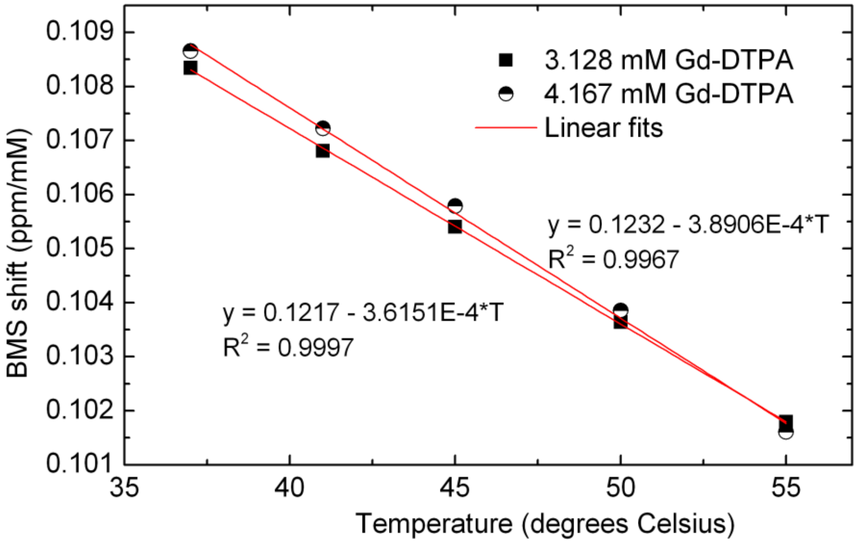

Supplemental figure 4: The BMS shift of the Gd-DTPA containing samples plotted against temperature. The slope of the linear fits represents the temperature dependency of the Gd-DTPA susceptibility ($d\Delta\chi_{Gd\text{-}DTPA}/dT \approx -0.00038 \pm 0.00008$ ppm/mM/°C).

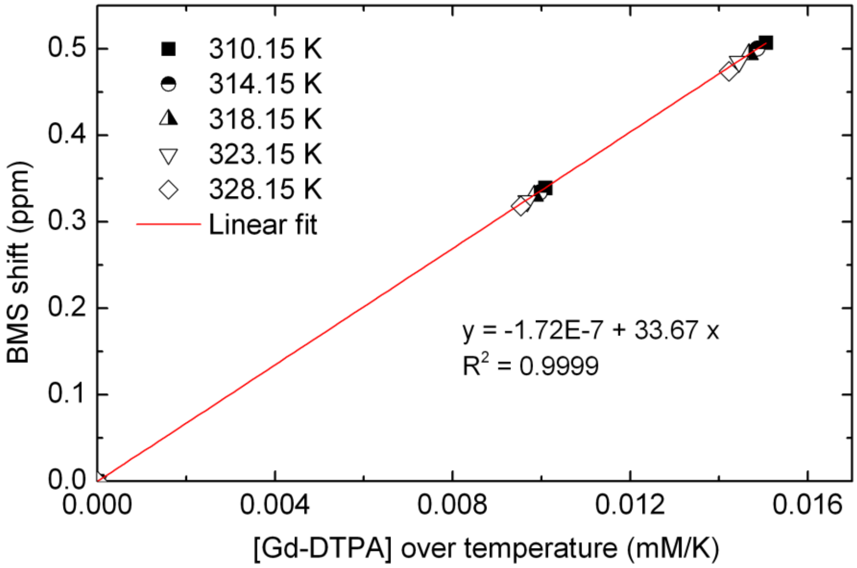

Supplemental figure 5: The absolute BMS shift plotted against the Gd-DTPA concentration over temperature in Kelvin. The resulting values of Δχ showed a linear correlation with the concentration-to-temperature ratio. Fitting with a linear model function yielded an excellent fit with an abscissa of almost zero and a slope of 33.67 ppm·K·mM^{-1} (R^2 = 0.9999), corresponding closely to the theoretical value, $4\pi s(\mu_{eff}/2.84)^2$ = 32.74 ppm·K·mM^{-1}, where s = 1/3 the shape factor for a cylinder parallel to the main magnetic field, and μ_{eff} = 7.94 for gadolinium (III) [1].

References

[1] Corsi DM, Platas-Iglesias C, van Bekkum H, Peters JA. Determination of paramagnetic lanthanide(III) concentrations from bulk magnetic susceptibility shifts in NMR spectra. Magnetic Resonance in Chemistry. 2001;39:723-6.
